# Quantum Pharmacophore-Based Virtual Screening Enables Prospective Discovery of Chemotype-Diverse Dengue NS5 Inhibitors

**DOI:** 10.1021/acs.jcim.6c01051

**Published:** 2026-07-16

**Authors:** Martin N. Martinov, Annelies Van Den Bergh, Edgar Jacoby, Ivaylo Kirov, Lyubomir G. Nashev, Oleksandra Herasymenko, Cheryl H. Arrowsmith, Jeffrey W. Slater, Tsehai Grell, Kenneth Maksimchuk, Michael D. Hack, Zhe Wu, Donya Ohadi, Doortje Borrenberghs, Marnix Van Loock, Chandrika Mulakala, Olivia Goethals

**Affiliations:** † Gradient Biomodeling, Park City, Utah 84098, United States; ‡ FAR Biotech, Madison, Wisconsin 53719, United States; § 6808 Johnson & Johnson Research and Development, Beerse, Antwerp 2340, Belgium; ∥ Gradient Bulgaria, Sofia 1164, Bulgaria; ⊥ Structural Genomics Consortium, University of Toronto, Toronto, Ontario M5G 1L7, Canada; # Johnson & Johnson Research and Development, Spring House, Pennsylvania 19477, United States; ∇ Johnson & Johnson Research and Development, San Diego, California 92121, United States; ○ Johnson & Johnson Research and Development, South San Francisco, California 94080, United States

## Abstract

In this work, we introduce a quantum pharmacophore framework that fundamentally redefines how molecular interactions are represented and screened computationally. Unlike traditional pharmacophore or docking-based approaches that rely on empirical feature definitions, conformational sampling, and Cartesian coordinates, our method derives pharmacophores directly from density functional theory and quantum theory of atoms in molecules. This yields a target-conditioned, topological, and interaction-centric representation of ligand–target complexes, enabling rigorous dimensionality reduction and substantial computational acceleration. To our knowledge, this is the first demonstration of a quantum-derived topological pharmacophore capable of supporting subgraph isomorphism-based virtual screening at chemical library scale. We prospectively applied quantum pharmacophore-based screening to three conserved pockets of the dengue virus NS5 RNA-dependent RNA polymerase, evaluating 43.8 million compounds and identifying five chemically diverse inhibitors validated in biochemical, biophysical, and cellular assays. Notably, three compounds target the highly dynamic NITD-640 pocket with biochemical IC50 values in the low- to mid-micromolar range (∼3 to 123 μM), improved ligand efficiency, and drug-like properties relative to the reference ligand. Together, these results demonstrate that quantum pharmacophores can uncover dissimilar chemotypes at challenging, flexible binding sites that are poorly addressed by conventional screening methods.

## Graphical abstract

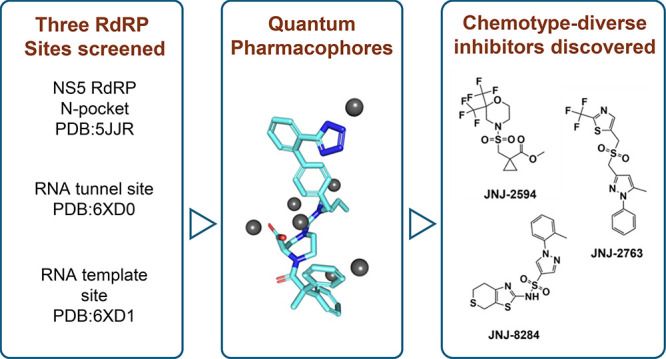

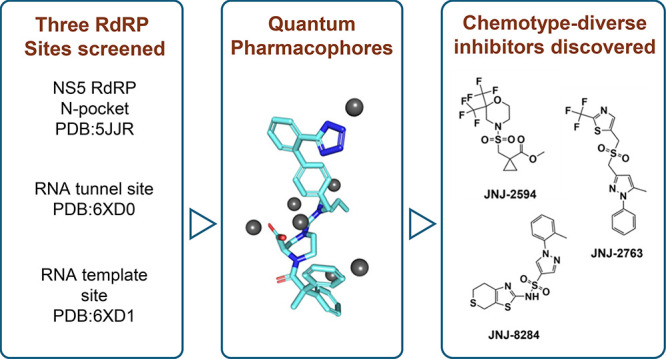

## Introduction

Paul Ehrlich is credited with originating the concept of the pharmacophore over a century ago, describing it as the spatial patterns of abstract molecular features that are ultimately responsible for the biological effect.^1^ Traditional pharmacophores are defined by the IUPAC as “the ensemble of steric and electronic features that is necessary to ensure the optimal supra-molecular interactions with a specific biological target structure and to trigger, or to block, its biological response”.^2^

Structure-based pharmacophores distill explicit all-atom representations of protein–ligand complexes into a set of key interactions. This abstraction parallels dimensionality reduction in data analysis and machine learning (ML) — a computational cost-saving technique that transforms high-dimensional data into a lower-dimensional space while preserving essential features. Similarly, a pharmacophore, at the right level of abstraction, offers a computationally efficient, lower-dimensional representation of biologically relevant protein–ligand interactions. Moreover, this representation naturally lends itself to the computational scale essential for virtual screening (VS).^3,4^

Traditional pharmacophoric features — including hydrogen bond acceptors, hydrogen bond donors, hydrophobic motifs, and aromatic groups — encode electronic interactions in a simplified, often imprecise form. Here, we present a rigorous generalization of such electronic interactions within the theoretical formalism of density functional theory (DFT)^5^ and quantum theory of atoms in molecules (QTAIM).^6^ As discussed in further detail below, mathematical attributes of the electron density field are directly related to localized molecular interactions and can be applied to a protein–ligand complex. Gradient Biomodeling’s (GB) quantum pharmacophores (QPs) are defined through molecular representations via energy-based quantum components. Following a systematic procedure (Figure 1A), we demonstrate that the resulting QP, when used for VS of conserved pockets of the Dengue virus Nonstructural 5 (NS5) RNA-dependent RNA polymerase (RdRP) protein, extrapolates to hits with vastly different chemical structure when compared to the starting chemical matter.

**Figure 1:**
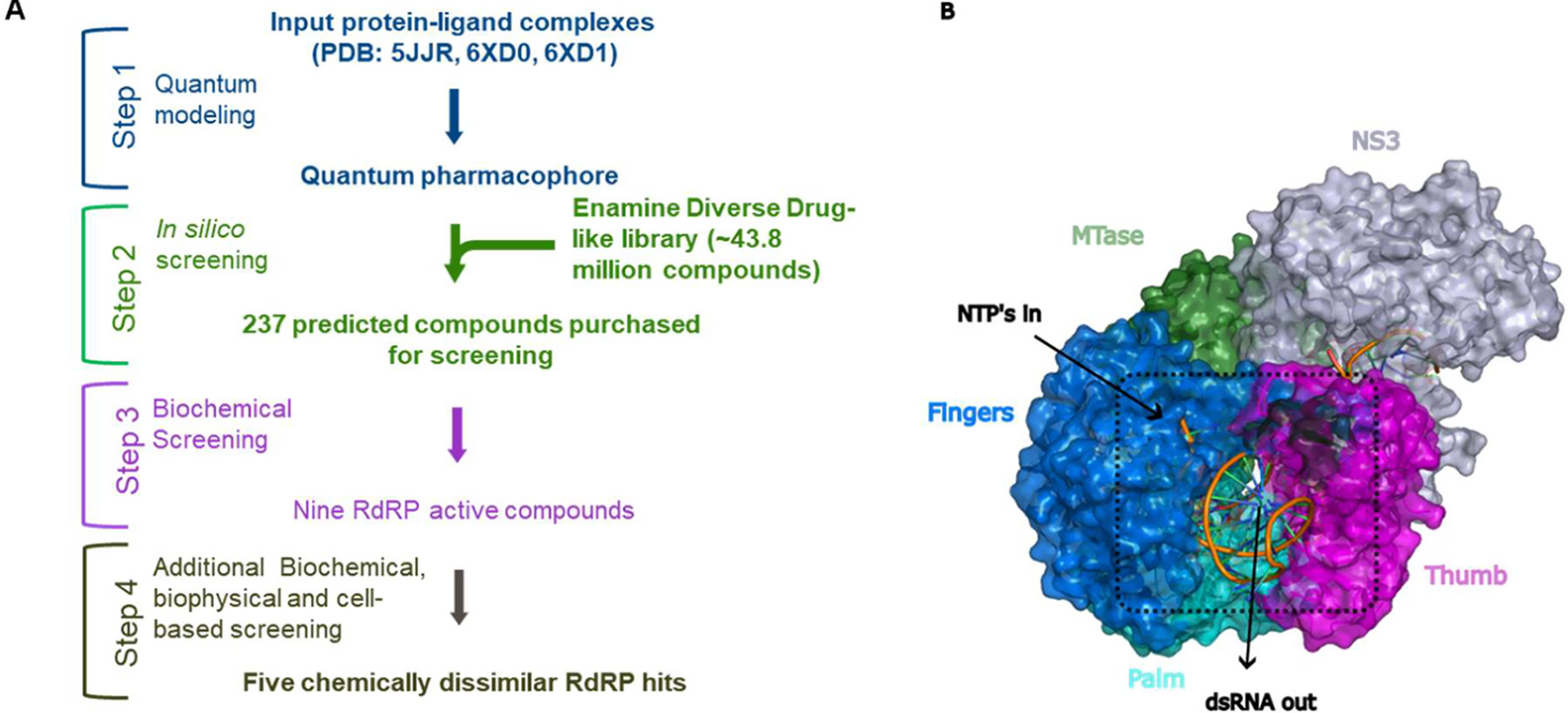
Representation of the QP-based VS process of DENV NS5 protein. (A) QPs were developed for three distinct binding pockets and their ligands within the RdRP structure. These QPs were then used to screen the Enamine Diverse Drug-like REAL library. Next, the top-ranked compounds were tested in DENV biochemical elongation assays, and active compounds were further tested in additional biochemical, biophysical, and cell-based assays for their antiviral activity. (B) Surface representation of the DENV3 NS5-NS3 complex (PDB ID: 8GZR): the fingers, thumb, and palm subdomains of the RdRP are colored blue, pink, and cyan, respectively. The active site is highlighted with a dashed box, showing NTP entry and dsRNA exit.

These underlying molecular representations have also been previously applied in a fuzzy decision network implementation for molecular similarity modeling utilizing ligand data sets (ligand-based pharmacophore screening) in the absence of structurally resolved macromolecular targets. The resulting methodology has been shown to require smaller data sets for training and has been experimentally validated against diverse therapeutic targets.^7–10^

While several quantum-derived interaction frameworks exist—such as QM/MM interaction energy pharmacophores,^11,12^ QTAIM-based interaction topology,^13,14^ FMO interaction fingerprints,^15,16^ and quantum similarity metrics^17,18^ —none provide a topological, target-conditioned quantum representation that can be leveraged for subgraph-isomorphism virtual screening at chemical library scale, as enabled by our quantum pharmacophore (QP) formalism.

Dengue viruses belong to the *Flaviviridae* family, which also includes West Nile virus (WNV), Zika virus (ZIKV), Yellow Fever virus (YFV), and Japanese Encephalitis virus (JEV). This vector-borne virus circulates as four dengue serotypes (DENV1–4) in tropical and subtropical countries, and all four can infect humans through mosquito bites.^19^ Dengue virus infection most commonly leads to a mild fever that resolves on its own, but can progress to ‘dengue fever’ or ‘severe dengue’, which is characterized by high fever, intense pain, and requires hospitalization.^20^ The World Health Organization (WHO) estimates that approximately 96 million people are infected with dengue virus annually, with an estimated 40,000 deaths attributed to dengue fever.^21^

The dengue virus genome is positive-sense, single stranded RNA that consists of seven open reading frames encoding a single polyprotein. Co- and post-translational processing by viral and host proteases is required to release the structural proteins (C, prM, E) and nonstructural proteins (NS1, NS2A, NS2B, NS3, NS4A, NS4B, NS5).^22^ Across all flaviviruses, NS5 is the most conserved protein, exhibiting a high level of homology between DENV, ZIKV, JEV, YFV, and WNV. NS5 is a multifunctional enzyme critical for viral replication and comprises two domains: an N-terminal methyltransferase (MTase) domain and a C-terminal RdRP domain. NS5 serves as the cornerstone of the replication complex, functioning in coordination with NS3.^23,24^ NS3 is critical for its helicase activity to unwind the de novo RNA that is synthesized by NS5 (Figure 1B). The RdRP exhibits primer-independent activity and initiates RNA synthesis de novo. Upon primer recognition — also referred to as initiation — conformational changes occur within the RdRP active site to facilitate primer elongation into nascent double stranded RNA. Within this framework, motifs E, F, and G play key roles in RdRP function and flexibility: motif F at the RNA template entrance undergoes pronounced ordering upon ligand binding (e.g., with NITD-640), motif G contributes to conformational changes adjacent to the active site during template engagement, and motif E supports positioning of catalytic elements during initiation and elongation. These conformationally dynamic motifs help define conserved, drug-addressable pockets that we target in this work.

The RdRP conformation resembles a right-hand, comprising fingers (residues 272–493, 540–599), palm (residues 494–539, 600–710), and thumb (residues 711–789, 805–900) subdomains. These form motif regions that bind tRNA and synthesize packaging RNA (pRNA) by linking nucleoside triphosphates (NTPs) at the Glycine-Aspartate-Aspartate (GDD) catalytic side, as highlighted in Figure 1B.^25^ Host cells typically lack enzymes required to generate double-stranded (ds)­RNA, making viral RNA polymerases attractive drug targets — as demonstrated by HIV-1 Reverse Transcriptase, HBV, CMV, HCV.^26^ Numerous drug discovery campaigns have targeted DENV RdRP to identify non-nucleoside small molecule inhibitors, with several hits identified in either homologous RdRP pockets or novel sites.^27–32^ However, none have progressed to clinical development due to lack of cellular or in vivo activity, high lipophilicity, and exhausted SAR — highlighting the challenges of targeting RNA-binding pockets with drug-like small molecules.^28,29^

Herein, we apply GB’s novel and precise structure-based QP approach to identify new chemical matter in three functional and conserved pockets within the NS5 RdRP.^27,28^ We identified five compounds which have strong drug-like potential and inhibit both the RdRP activity and cellular DENV2 replication.

## Theoretical Background on Quantum Pharmacophores

GB’s proprietary computational methodology combines concepts from DFT and QTAIM to define QPs based on electron density calculations which generate quantum representations of molecules and their interactions. Consequently, instead of treating molecules as sets of bonded atoms, GB’s approach captures them as topological manifolds at the fundamental level of electron density. These manifolds are derived from chemical structures but are not directly dependent on their underlying atomic configurations. Since distinct atomic configurations can correspond to similar manifolds, this implicit, rather than explicit, relationship between QPs and underlying atomic configurations enables the identification of in silico hits that often diverge significantly from the reference ligand, enabling the identification of novel active compounds.

Since biochemical molecular interactions are quantum in nature, nonrelativistic quantum mechanics presents the proper level of physical theory for their mathematical modeling.^33^ Within this theoretical framework, DFT rigorously introduces general chemical concepts through the use of the electron density, ρ­(**r**), defined in eq 1, a real, nonnegative Cartesian function related to the *N*-electron molecular wave function Ψ:

$$\rho \left(r\right)=\int |\Psi ({x,x_{1},\cdot \cdot \cdot ,x_{N-1})|}^{2}\mathrm{dsd}x_{1}\;\mathrm{...},dx_{N-1}$$ 1

where **x** = {*s*, **r**} is the four-dimensional spin-spatial coordinate. As the fundamental Hohenberg–Kohn theorem shows, ρ­(**r**) determines all ground-state properties of the entire system, including its chemical and biochemical features. Furthermore, QTAIM uses ρ­(**r**) to partition molecules into precise, interconnected atomic subsystems. These atomic subsystems are bounded by zero-flux surfaces **S**, as defined in eq 2:

$$\forall_{r\in S}n\left(r\right)•\nabla \rho \left(r\right)=0$$ 2

where **n**(**r**) is the vector normal to **S** at **r** and ρ­(**r**) is the corresponding electron density. These so-defined atomic basins are topologically stable and even allow transferability between molecular systems with similar adjacency.^34^

The ability to naturally decompose the electron density field of molecular systems into rigorously defined quantum subsystems with underlying incidence structure is a powerful tool for their investigation. Since molecular interactions are contained within a topological object, there exists no need for conformational sampling or any other operations in Cartesian space. Instead, the problem of matching the interaction pharmacophore to prospective compounds can be defined as a subgraph isomorphism procedure. Even though this presents its own computational challenges,^35,36^ multiple practical solutions exist, including some that benefit from systematic applications of ML as further described below.

Furthermore, when combined with molecular property definitions from DFT, this approach can be used to rigorously delineate and calculate multiple localized molecular properties by analyzing the resulting self-contained, chemically relevant, atomic regions of the electron density field. This combination has yielded meaningful interpretations of various physicochemical concepts, such as molecular energy decomposition,^37^ atomic softness,^38^ electronegativity equalization,^39^ atomic reactivity indices,^40^ and others.^41,42^

Of particular interest here is the energy decomposition methodology described in Martinov et al.^37^ To account for systems of chemical and biological interest, the interaction energy is defined, as shown in eq 3, as the difference between the energy of a supermolecular complex and the sum of the energies of the isolated molecular subsystems *A* and *B* forming the complex:

$${Ε}_{\mathrm{int}}={Ε}_{AB}-{Ε}_{A}^{0}-{Ε}_{B}^{0}$$ 3

This formalism allows the use of DFT to compute the energy lowering due to the electronegativity equalization that occurs when a supermolecule is formed from its constituent subsystems (Figure 2A). These subsystems are rigorously defined by QTAIM. Specifically, the number of electrons within subsystem *A* is given by eq 4.

$$N_{A}=\int_{\Omega_{A}}\rho (r)dr$$ 4

where Ω_*A*_ is the union of the atomic basins comprising the subsystem *A* of the supermolecule. The total charge of *A*, *Q*_*A*_, is then equal to the difference between the respective total nuclear charge, *Z*_*A*_ and *N*_*A*_. The energy difference related to the electronegativities of *A* and *B* in situ presents a measure of their partial interaction within the supermolecule,^5,43,44^ as defined in eq 5:

$$E_{CT}=E_{AB}\left(Q_{CT}^{0}\right)-E_{AB}\left(0\right)$$ 5

where

$$Q_{CT}=Q_{A}-Q_{A}^{0}$$ 6

**Figure 2:**
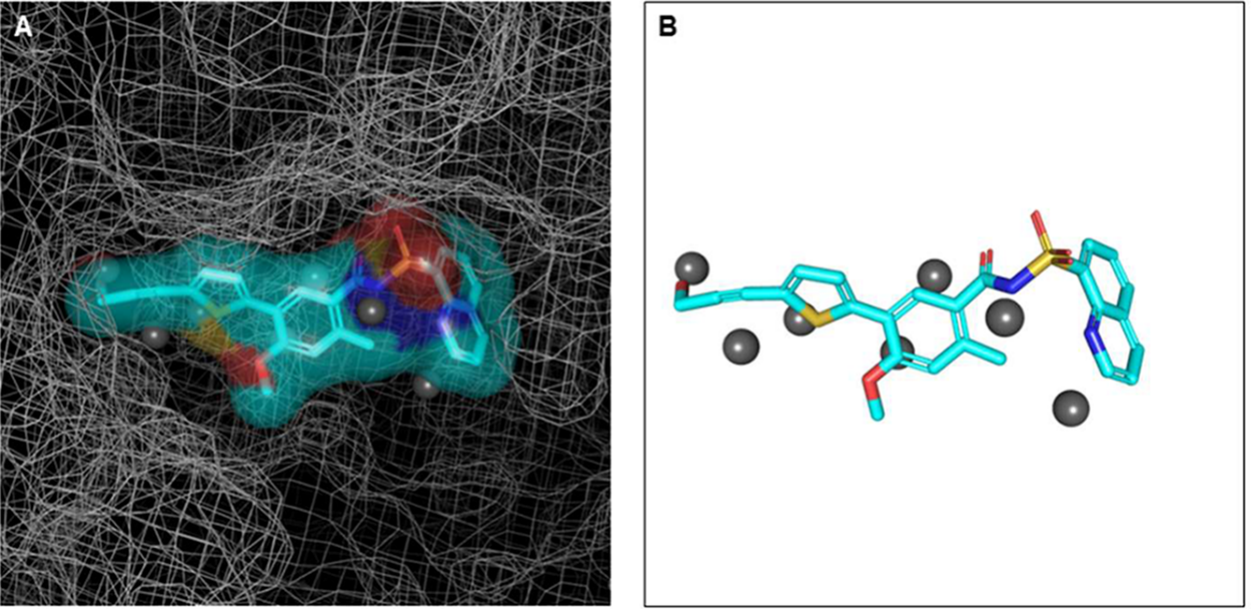
Calculation of QPs. (A) QP of the interaction within the complex of DENV2 RdRp (gray mesh) and the inhibitor compound 29, which binds to the N-pocket of the RdRP^28^, is presented as gray spheres situated at the protein–ligand interface region (PDB 5JJR). The ligand is shown as color-coded sticks covered by a transparent surface. (B) Stand-alone QP of the interaction of compound 29 with NS5. Compound 29 is shown in the same orientation as in 2A. The gray spheres represent activity-controlling, electron-density based local interactions, as described in greater detail in the theoretical background on QPs.

eq 6 defines the charge transfer, *Q*_*CT*_, between the two subsystems *A* and *B*. The derivative,

$$\lambda \left(Q_{CT}\right)=\frac{\partial E_{AB}}{\partial Q_{CT}}$$ 7

as defined in eq 7, describes the charge-transfer driving force and is used as a Lagrange multiplier in the calculation of its localized interaction energy component. Since this formalism does not rely on Hilbert space partitioning, the resulting interaction energy terms are well-defined true observables converging smoothly with the completeness of basis sets.^37^

The general computational strategy has been described previously.^37^ Conceptually, it can be formulated in second-quantization terms independently of the chosen electronic-structure approximation. Starting with the QTAIM number-of-electrons operator (eq 4), we have eq 8:

$${\hat{N}}_{A}=\sum_{i}\int_{\Omega_{A}}\delta (r_{i}-R)dR$$ 8

the second-quantized Hamiltonian is given by eq 9:

$$\hat{H}=\sum_{ij}h_{ij}{\hat{a}}_{i}^{+}{\hat{a}}_{j}+\left(\frac{1}{2}\right)\sum_{ijkl}<ij|kl>{\hat{a}}_{i}^{+}{\hat{a}}_{j}^{+}{\hat{a}}_{l}{\hat{a}}_{k}$$ 9

can be modified to reflect electron flow (eq 7) yielding the formulation in eq 10:

H^[λ(QCT)]=∑ij[hij+λ(QCT)nij]a^i+a^j+(12)∑ijkl<ij|kl>a^i+a^j+a^la^k 10

where {*â*_*i*_^+^} and {*â*_*i*_} denote the creation and annihilation operators. Further, {*n*_*ij*_} define and can be computed as the *N̂*_*A*_ matrix elements. When combined with the established capability of QTAIM to derive incidence structures from the molecular Laplacian fields,^45^ this formalism avoids the computational overhead associated with direct electron density manipulation. Importantly, the resulting QTAIM-derived incidence structures can be represented as spatial interaction networks.^14,45^

Translating the underlying physical interactions into such framework can leverage machine-learning methodologies already developed for spatial network analysis since the spatial incidence matrix $B\in R^{\left|V\right|\times \left|E\right|}$ provides an efficient algebraic encoding of the quantum interaction topology and permits scalable graph traversal, spectral decomposition, and subgraph-matching operations.^46,47^

In general, spatial network applications define $B\in R^{\left|V\right|\times \left|E\right|}$ as shown in eq 11.

$$B_{\mathrm{ve}}=\{\begin{array}{c} w_{e},\mathrm{if}\;\mathrm{node}\;v\;\mathrm{is}\;\mathrm{incident}\;\mathrm{to}\;\mathrm{edge}\;e\\ 0,\mathrm{otherwise} \end{array}$$ 11

where {*w*_*e*_} are edge weights, which typically depend on Cartesian metrics and other problem-specific properties. Spatial network representations are widely used across various scientific domains and are increasingly adopted in computer-aided drug design, where {*w*_*e*_} typically incorporate discrete molecular descriptors in addition to Euclidean distances.^48–50^

In contrast, our developed workflow (Figure 3) is executed natively within the topological space. Operating in this abstract space allows the framework to evaluate interactions based on core electronic relationships rather than rigid geometric constraints. Thus, by projecting the system into this space, the identification of viable binding partners is streamlined into a weighted subgraph isomorphism calculation. To solve this efficiently, we have designed a proprietary machine learning architecture optimized specifically for rapid subnetwork matching (Figure 3 and discussion below).

**Figure 3:**
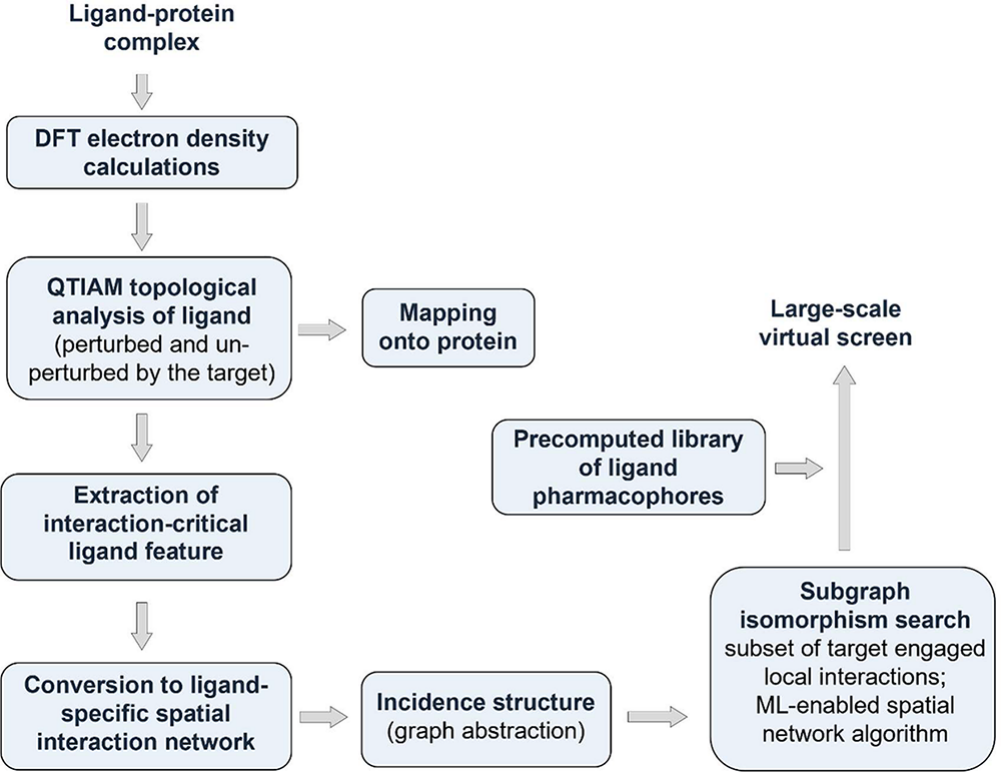
Quantum pharmacophore-based virtual screening workflow. Ligand–protein complexes are first represented at the level of electron density using density functional theory (DFT), followed by quantum theory of atoms in molecules (QTAIM)–based partitioning to generate a target-conditioned topological description of the ligand. Extraction of interaction-critical ligand features and conversion into a ligand-specific spatial interaction network yield an incidence structure (graph abstraction) that encodes the underlying quantum interaction topology. The resulting quantum pharmacophore (QP), defined as a subgraph of target-engaged interaction motifs, is used as a query for large-scale virtual screening. Efficient matching against a precomputed library of ligand pharmacophores is performed via subgraph isomorphism search in topological space using machine learning–enabled graph algorithms, enabling rapid identification of chemically diverse hits without reliance on conformational sampling or Cartesian coordinate alignment.

The above energy-decomposition procedure and associated spatial network analysis can be generalized and applied to structurally resolved proteins cocrystallized with a small molecule. The target-ligand complex is regarded as a supermolecule, and the local interactions within the complex are computed as a ligand-specific QP model (Figure 2B). Since the topological analysis described above presents the QP as a network based on the ligand-target supermolecule, it can be also compared to the native network of the free ligand. Such a comparison indicates which electronic features of ligand drive the interaction with the target.

Within this topological formulation, the target acts as a perturbation on the free ligand. To improve computational efficiency, target-related calculations can be performed at a lower level of theory without compromising the topological representation. Generating the ligand-specific incidence structures remains the dominant computational cost, particularly for large ligands such as biologics or small proteins. For small molecules, native ligand networks can be precomputed and stored, enabling efficient large-scale screening of large chemical spaces.

Overall, this approach employs a highly customized DFT/QTAIM framework to analyze ligand-specific electron-flow patterns in biomolecular systems. The resulting incidence structures—computationally analogous to spatial networks—provide an interpretable mapping of the electron redistribution induced by target binding. Dynamic binding pockets, which are often challenging for conventional docking approaches, are effectively addressed within this framework, as the QP representation captures conserved interaction topology that remains robust to protein flexibility.

In summary, QTAIM enables the construction of molecular incidence-structure representations that can be processed using machine-learning-enhanced spatial-network algorithms,^14,45^ This reduces the search problem to subgraph-isomorphism queries within precomputed databases spanning large chemical spaces. The above energy-decomposition procedure can be generalized and applied to structurally resolved proteins cocrystallized with a small molecule. The target-ligand complex is regarded as a supermolecule, and the local interactions within the complex are computed as a QP model (Figure 2B). Since the topological analysis described above presents the QP as a network based on the ligand-target supermolecule, it can be also compared to the native network of the free ligand. Such a comparison indicates which electronic features of ligand drive the interaction with the target.

## Computational Efficiency of GB’s Methodology

A key feature differentiating QPs from traditional pharmacophores stems from their first-principles derivation, which allows rigorous treatment of the pharmacophores as molecular networks. In graph-theoretical terms, this means that the interaction pharmacophore is derived as a subgraph of the ligand pharmacophore, which is then applied in the search for prospective novel ligands.

One substantial computational advantage comes from the fact that complete ligand pharmacophores for entire chemical search spaces can be precomputed and stored in a database for fast and efficient screening. This is computationally expensive with traditional pharmacophores due to the need for ligand conformational sampling and Cartesian alignment. As described above, this VS methodology leverages the topological properties of ligand-target interaction QPs and does not rely on docking in Cartesian space. In general, current docking algorithms have difficulties in handling ligands with considerable conformational freedom, especially when simultaneously addressing target flexibility.^51^ Moreover, since the QP represents the protein–ligand complex at a topological level — without requiring an explicit coordinate-based model of the target — these computational efficiencies naturally extend to the conformational sampling of the target as well.

Further, traditional pharmacophore models require the specification of exclusion volumes to delineate regions within the binding site that ligands must avoid due to potential steric clashes with the receptor. This necessity arises from the fact that traditional chemical pharmacophoric features form a discrete set, without any geometric properties. In contrast, QPs are derived from molecular graphs, which already contain bond lengths and other geometric constraints. Thus, QPs can be treated as spatial networks for which Cartesian — including volumetric — properties can be described with simple parameters and incorporated seamlessly in the VS procedure^52^ (Figure 3). As discussed above, this abstraction substantially accelerates screening because graph-based matching scales far more efficiently than repeated comparison of full electron-density volumes or iterative quantum recalculations. Importantly, the framework preserves the interaction topology underlying molecular recognition while reducing sensitivity to conformational variability, thereby enabling the identification of electronically analogous yet chemically diverse scaffolds. As a result, this approach bypasses the limitations of traditional, geometry-bound computational schemes. Since GB’s algorithm captures invariant electronic profiles rather than physical contours, it successfully identifies chemically novel, nonobvious molecular frameworks that share identical functional mechanisms despite having entirely distinct structural backbones.

To summarize, performance optimization in GB’s approach occurs at several levels. First, the QP is derived from the protein–ligand complex and contains only the subset of ligand quantum attributes relevant to its interaction with this particular target. Thus, the search for prospective novel ligands can be addressed as subgraph matching. This provides significant computational acceleration by simultaneously reducing the dimensionality of the problem and eliminating the need for conformational sampling. Second, the complete pharmacophores for entire chemical search spaces of prospective ligands can be precomputed and stored in a database for fast and efficient screening. Finally, since the quantum pharmacophores are generated and used for screening in an optimal non-Cartesian space, GB’s approach significantly outperforms conventional docking while maintaining a high diversity of chemical classes among the identified hits. In practice, by leveraging these computational efficiencies, QP-based VS workflows can be executed in a matter of days on standard hardware (Intel CPU@3.00 GHz, 72 logical processors, 128 GB RAM, 1 TB NVMe SSD). This demonstrates that more complex quantum models do not necessarily compromise computational efficiency and can even offer superior scalability for modern drug discovery pipelines.

## Results

### QP Modeling of DENV RdRP

Three structurally conserved and functionally relevant DENV RdRP pockets were selected for QP modeling. The first pocket, known as the N-pocket, was chosen because of its high conservation across several flaviviruses,^21^ including JEV, WNV and YFV within 5 Å of the pocket. Moreover, this allosteric pocket adapts different conformations shaped by the priming loop in *apo*, ligand- and substrate bound structures. Lim et al. were the first to highlight the functional importance of the DENV RdRP N-pocket through a fragment-based X-ray crystallography screen. The initial fragment found in the pocket was optimized through rational design into a nanomolar RdRP inhibitor, compound 29. Therefore, PDB ID 5JJR was chosen for modeling as it is the co-complexed structure of compound 29 in the N-pocket resolved up to 1.99 Å.^28^

The second screening site lies within the RNA tunnel, between the fingers and palm subdomain, and in close proximity to the active site. To date, three inhibitors—NITD-434, NITD-107 and HeE1–2Tyr—have been reported to bind within this pocket.^27,29,53^ The pocket is also highly conserved within the flavivirus family and different conformations between the *apo* and ligand-bound states have been noted, particularly of motifs B and G. Since NITD-434 is the most potent inhibitor identified to date for this pocket, its cocrystal structure (PDB ID 6XD0) was selected as the second screening site.

The third site is located near the entrance of the RNA template within the fingers subdomain. Thus, far, only one ligand bound structure has been described: PDB ID 6XD1, where NITD-640 with moderate polymerase inhibition activity, stabilizes motif F. This flexible and mobile motif is commonly unresolved in the majority of RdRP structures and was selected as the final screening site.^27^

A comparison of GB’s QPs of DENV NS5-inhibitor interactions with traditional pharmacophores is shown on Figure 4. The QPs (panels B, C, E, F, H and I, Figure 4) illustrate electron-density based local interactions derived from first-principles (gray spheres). The traditional pharmacophore view (panels A, D and G, Figure 4) depicts potential local interactions with the protein target, including hydrogen bond acceptors/donors, aromatic rings, and hydrophobic interactions (colored spheres). A comparison of the left and middle panels of Figure 4 clearly demonstrates that GB’s method identifies interactions that differ significantly from those represented in traditional pharmacophores, thereby enabling the identification of hits that are markedly distinct from the starting chemical matter, without compromising accuracy. For VS, the local interactions in the central panels (QPs) were used as the subgraph for each site to search for novel ligands with improved subgraphs and thus improved interactions with the target compared to the reference ligand from which the pharmacophore is derived.

**Figure 4:**
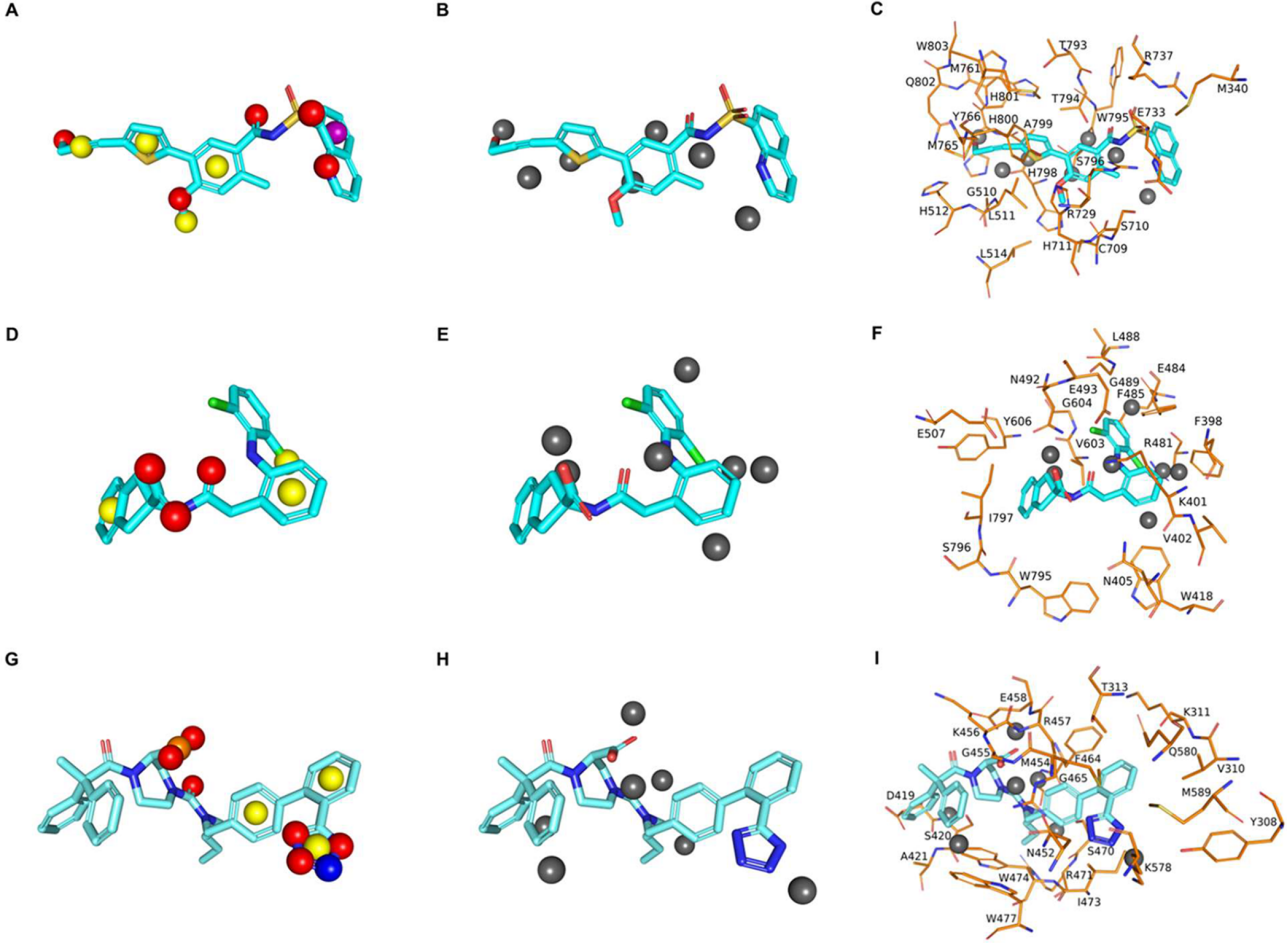
Quantum versus traditional pharmacophores. (A–I) Models based on PDB 5JJR; (A) traditional pharmacophore model of NS5 – compound 29 interaction, generated with PharmIT.^54^ Hydrogen bond acceptors are depicted as red spheres, hydrogen bond donors as blue spheres, hydrophobic interactions are in yellow, and aromatic rings in purple. (B) QP model of NS5-compound 29 interaction. Local interactions between ligand and target are defined from density-functional concepts and displayed as gray spheres; (C) QP and neighboring amino-acids of the protein target, represented as sticks (carbons in orange). Residues located within 5 Å from the ligand are shown. (D–F) Models based on PDB 6XD0, representing the second NS5-binding site; (D) traditional pharmacophore of the interaction of NS5 and inhibitor NITD-434; (E) QP; (F) QP and neighboring amino-acids of the target. Pharmacophore elements and color coding are the same as in A. (G–I) Models based on PDB 6XD1 (NS5 – NITD-640 interaction); (G) traditional pharmacophore; (H) QP; (I) QP including neighboring protein-target residues. Color-coding is as in C and F.

### Scoring of Novel Chemical Matter against Three Sites within DENV RdRP

Employing the procedure outlined in Figure 2A, GB generated QPs for all three binding sites using PDB IDs 5JJR, 6XD0 and 6XD1 (Figure 4). Each QP was used in a separate VS against the Enamine REAL Diverse Drug-like library, which contained ∼43.8 million compounds at the time these screens were conducted.

Since the objective of these screens is to identify compounds for procurement and testing, the screening protocol was defined as a binary classification problem. Accordingly, the quantum scores of the respective cocrystal ligands—when computed and used as thresholds—served as an algorithmically efficient and strong necessary condition for the activity of candidate hit compounds. GB’s general prioritization procedure also includes the aforementioned Cartesian constraints, a requirement for low structural similarity to the corresponding cocrystal ligand (Tanimoto coefficient using ECFP fingerprints <0.5,^55^ and cheminformatic and medicinal chemistry considerations) good drug-likeness properties and minimal presence of undesirable chemical groups.^56^ GB’s protocol is therefore fully automated, with no human input biasing the results.

Using the criteria mentioned above, model 5JJR yielded 137 virtual hits with scores exceeding the threshold defined by the respective cocrystal ligand.^57^ The 6XD0 model yielded 138 virtual hits, while the 6XD1 model generated 130. These compounds were selected for procurement and biochemical screening. Of the total 405, only 237 were available for purchase from Enamine, which were subsequently tested in a biochemical assay.

### Hit Identification through Target-Based Assays

To assess RdRP activity, the 237 purchased compounds (∼80 for each site) were evaluated using a biochemical polymerase assay with DENV2 and DENV3 RdRP proteins. Full dose–response curves were generated starting at 100 μM for all 237 compounds. Nine compounds with half maximal inhibitory concentration (IC_50_) ≤ 100 μM were identified as active against both DENV2 and DENV3 NS5. These hits were further characterized for their binding affinity to DENV2 RdRP using an surface plasmon resonance (SPR) assay, resulting in five candidates that showed activity in both biochemical and biophysical assays (Figure 5, Table 1) leading to hit rates of 1.3–4%.

**Figure 5:**
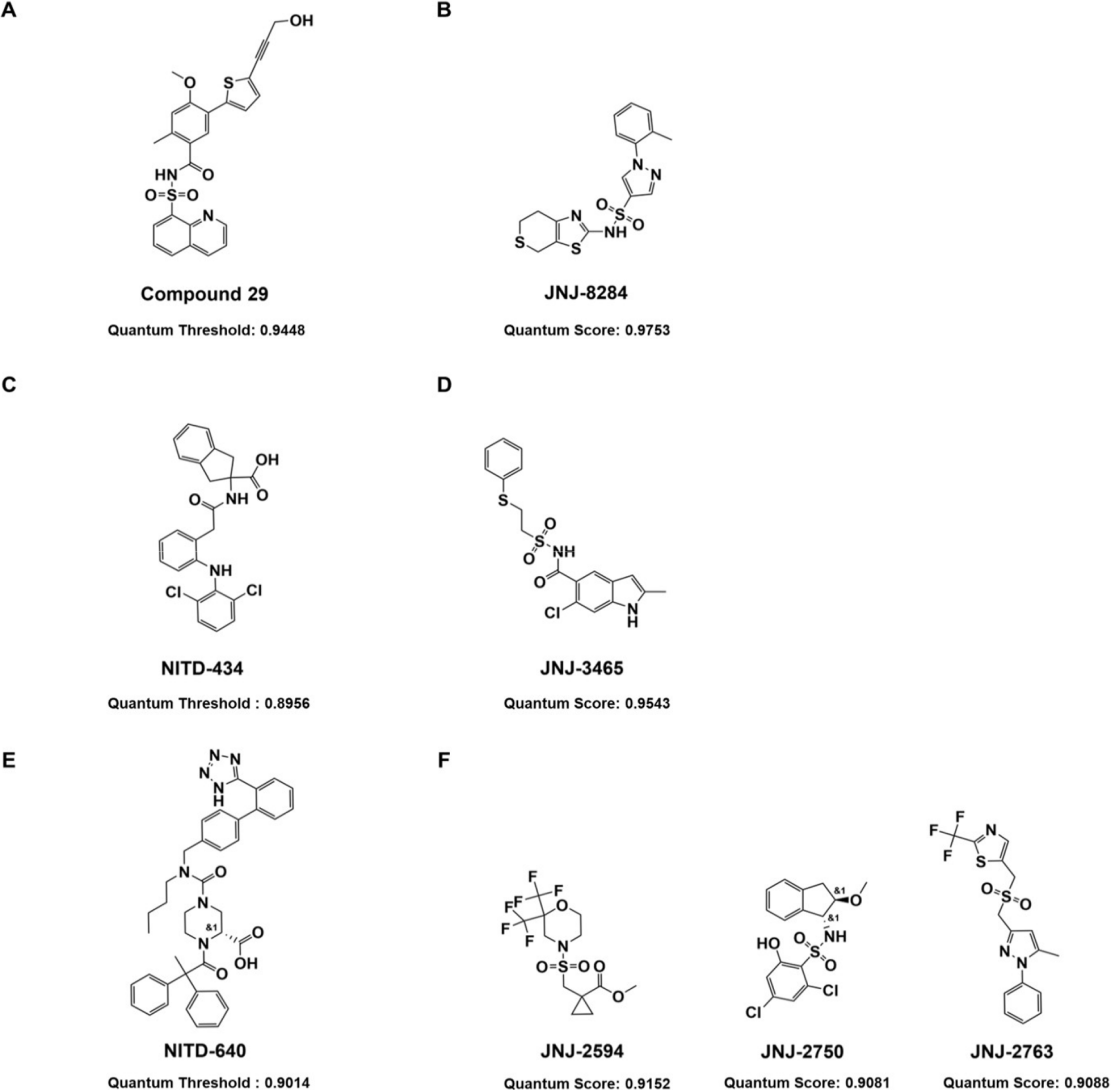
Structures of the identified hits compared to their reference ligands in (A, C, E). For each ligand, quantum scores are indicated below the structure and were used as the threshold to select virtual hits. Further, as surmised, the hits are markedly dissimilar from the reference ligands (A) Compound 29, cocrystal ligand of PDB 5JJR and (B) the hit compound JNJ-8284 (C) NITD-434 of PDB 6XD0 and (D) hit compound JNJ-3465. (E) NITD-640 of PDB 6XD1 and (F) the three hits identified in the VS.

**Table 1:** DENV Target Activity of Hits with Activity across Multiple Target-Based Assays^a^,^b^,^c^

pocket	compound	**elongation assay**		**SPR assay**		**RNA synthesis assay**	
		DENV2_NGC	DENV3_SL	DENV2_PG		DENV2_TVS01	h-mtRNA
		NS5 FL	NS5 FL	NS5 RdRP		NS5 FL (de novo)	polymerase (primed)
		*n* = 3	*n* = 3	*n* = 2–4		*n* = 2	*n* = 1
		**IC**_**50**_ **(μM)**	**IC**_**50**_ **(μM)**	***K***_**D**_ **(μM)**	***R***_**max**_ **(RU)**	**IC**_**50**_ **(μM)**	**IC**_**50**_ **(μM)**
5JJR	JNJ-8284	24.7 ± 29	25.7 ± 30	5.5 ± 1.3	177	39.4 ± 30.6	5.7
	*compound 29*	2 ± 0.8	0.02 ± 0.002	0.096 ± 0.052	40.9	2.95 ± 0.95	15.5
							
6XD0	JNJ-3465	139.1 ± 35	109.6 ± 15	22.6 ± 7.2	41.7	>100	NT
	*NITD-434*	>57.5	5 ± 0	17.8 ± 8.4	88.2	13.5 ± 0.5	102
							
6XD1	JNJ-2763	123	60.7 ± 7.3	34 ± 4	74.9	363	160
	JNJ-2750	93.3	83.2	linear response	/	57	92
	JNJ-2594	5.8 ± 1.3	3.2 ± 0.5	10 ± 2	249	23.5 ± 5.5	5.2
	*NITD-640*	59.2 ± 14	30.1 ± 4	12.0 ± 8.5	192	6.4 ± 1.3	60.5

a For each site, the reference ligand is included for comparison. The data represents the average IC_50_ values and average *K*_D_ from at least 2 independent repeats ± standard deviation, where applicable.

b If no standard deviation is reported, the value was calculated from only one dose-response curve.

c *K*_a_ and *K*_d_ values are provided in the Supplementary Table 1, *R*_max_: vmaximum response, RU: response units.

One hit identified from a VS of the allosteric N-pocket, JNJ-8284, inhibited both DENV2 and DENV3 RdRP with IC_50_ values of 24.7 ± 29 and 25.7 ± 30 μM, respectively, and with a *K*_D_ of 5.5 ± 0.94 μM. Another hit, JNJ-3465, that putatively binds to the RNA tunnel, exhibited IC_50_ values of 139.1 ± 35 and 109.6 ± 15 μM, and a *K*_D_ of 22.6 ± 5.9 μM to the DENV2 RdRP protein. The NITD-640 binding site VS generated three hits with varying activities. The most potent hit was JNJ-2594 with IC_50_ values of 5.8 ± 1.3 μM for DENV2 and 3.2 ± 0.5 μM against DENV3 and a *K*_D_ of 7.5 ± 2.1 μM. JNJ-2763 and JNJ-2750 inhibited DENV2 RdRP with IC_50_ values of 123.0 and 93.0 μM, respectively. For JNJ-2750 a linear response was noted in the SPR assay, while JNJ-2763 showed a *K*_D_ of 34.4 μM. Sensorgrams for each compound are provided in Supplementary Figure 1.

All five hit compounds (Table 1) were tested against DENV2_TVS01 in the de novo RNA synthesis inhibition assay. JNJ-8284 exhibited inhibition values comparable to those observed in the biochemical assay, with an IC_50_ of 39.4 ± 30.6 μM. JNJ-2594 remains the most potent inhibitor identified, with an IC_50_ of 23.5 ± 5.5 μM. Interestingly, JNJ-3465 did not show confirmed inhibition in the de novo RNA initiation assay, likely because its IC_50_ exceeded the highest concentration tested.

The compounds were also evaluated for cross-reactivity against the human mitochondrial RNA polymerase (h-mtRNAP). All compounds, including the reference controls, demonstrated some degree of activity against the h-mtRNAP (Table 1). This may indicate a potential off-target concern for further development; therefore, enhancing the antiviral specificity of these inhibitors would be a key objective in optimizing the next series of compounds. Overall, these hits are direct inhibitors of RNA synthesis which is encouraging for the future development of these compounds.

### Potency of Discovered Hits against DENV2 in a Cell-Based Assay

Next, we evaluated whether the five RdRP-active compounds inhibit DENV2 infection in cellular assays. The compounds were tested at a starting concentration of 100 μM in a 9-fold dilution series against a DENV2 eGFP reporter virus in both Vero and Huh7 cells, which are readily susceptible to DENV2 infection. Four of the hits demonstrated clear concentration-dependent inhibition of viral replication with favorable selectivity indexes (SI = CC_50_/EC_50_) (Table 2), indicating that the observed antiviral effects are not driven by compound toxicity and highlighting the potential of these drug-like (i.e., Lipinski’s rule-of-five compliant, Table 3) hits. The most potent RdRP inhibitor, JNJ-2594, did not exhibit cellular activity, which may be attributed to cellular metabolism or poor cell permeability. The N-pocket candidate, JNJ-8284, was less potent in cellular assays than compound 29, with a Vero EC_50_ of 27.9 ± 8.3 μM, compared to 5.3 ± 2.2 μM for compound 29. However, JNJ-8284 is not active in the Huh7 cells at the highest concentration tested. Compound JNJ-3465, with an EC_50_ value of 24 ± 1.7 μM, shows comparable inhibition to the reference molecule NITD-434, while having a SI in Vero cells, unlike the control. For the second RNA tunnel site, only JNJ-2763 demonstrates concentration dependent inhibition with an EC_50_ value of 9.1 ± 8 μM and a reduction in potency in the Huh7 cells and increased toxicity, which must be considered when progressing this compound. Compound JNJ-2750 has a SI of >3 in the Vero cells, but an SI ∼ 1 in Huh7 cells which raises concerns that need to be addressed during hit optimization. Dose response curves for the compounds are provided in Supplementary Figure 2.

**Table 2:** Cell-Based Antiviral Activity of the DENV Polymerase Hit Compounds^a^

JNJB	**DENV2/eGFP** **(Vero)**			**DENV2/eGFP** **(Huh7)**		
	(*n* = 3)			(*n* = 3)		
	**EC**_**50**_ **(μM)**	**CC**_**50**_ **(μM)**	**SI**	**EC**_**50**_ **(μM)**	**CC**_**50**_ **(μM)**	**SI**
JNJ-8284	27.9 ± 8.3	>100	>3.6	>50	>100	>2
compound 29	5.3 ± 2.2	>50	>9.4	3.7 ± 0.3	>50	>13.5
						
JNJ-3465	24 ± 1.7	>100	>4.1	12.3 ± 6.5	>100	>8.1
NITD-434	23 ± 4.6	29.2 ± 3.3	1.2	20.5 ± 2	>50	>2.4
						
JNJ-2750	17.1 ± 6.5	>50	>2.9	25 ± 2	22.3 ± 5.5	0.9
JNJ-2763	9.1 ± 8	>100	>11	39.8	>22.91	>0.6
JNJ-2594	>50	>100	>2	>61.7	>50	>0.8
NITD-640	22.8 ± 4	>50	>2.1	19 ± 4	>50	>2.6

a The data represents the mean values ± standard deviations from at least three independent experiments. EC_50_: 50% effective concentration, CC_50_: 50% cytotoxic concentration, SI: selectivity index (=CC_50_/EC_50_).

**Table 3:** Drug-Like Properties of the Ligands and Identified Hits

	MW	cLogP	TPSA	HBA	HBD	LE^a^
JNJ-8284	392.52	2.92	138.8	4	1	0.25
compound 29	492.57	3.59	142.2	5	2	0.31
						
JNJ-3465	408.92	4.05	112.7	3	2	0.21
NITD-434	455.33	5.67	78.4	2	2	0.23
						
JNJ-2750	388.27	4.29	84	3	2	0.22
JNJ-2763	401.43	1.82	101.5	4	0	0.23
JNJ-2594	399.31	2.78	81.3	5	0	0.30
NITD-640	671.79	7.39	135.6	6	1	0.12

a Ligand efficiency based on the DENV3 elongation assay. LE = 1.37 × pIC50/*N*, where *N* is the number of non-hydrogen atoms in the ligand.^57^ MW: Molecular Weight, *c*Log*P*: calculated log *P*: a computational estimate of a chemical compound’s lipophilicity, TPSA: Topological Polar Surface Area, a predictor of molecular permeability, HBA: number of Hydrogen bond acceptors, HBD: number of hydrogen bond donors.

Encouragingly, all the identified hits have lower molecular weight than the reference ligands, and—all except JNJ-8284—exhibit potencies comparable to or better than the reference ligands. These compounds conform to Lipinski’s Rule of Five and represent attractive starting points for further optimization (see Table 3). Moreover, with the exception of JNJ-8284, all identified hits also have higher ligand efficiencies^57^ compared to the reference molecule that served as the basis of the computed QP, making them promising candidates for medicinal chemistry-driven optimization. Notably, although compound 29 has higher ligand efficiency (LE) than JNJ-8284, it is not a hit from a high-throughput screen like NITD-434 and NITD-640, but rather the result of a medicinal chemistry optimization campaign^28^ of a fragment hit. Also important to underscore is that none of the hits possess undesirable chemical functionalities, such as the acidic groups present in NITD-434 and NITD-640, which can significantly reduce cell permeability. JNJ-2594 exhibits the highest LE (0.31) and may serve as a desirable starting point for further hit-to-lead optimization.

## Discussion

The theoretical advantages of GB’s first-principles-derived QPs at VS scale prompted a rigorous evaluation to assess their utility. Motivated by the novelty and potential impact of GB’s molecular representations, we first undertook a retrospective study using historical and proprietary data from J&J (not reported here) to examine the approach and compare it to a range of VS tools available at J&J, which included 2D substructure and similarity searches, 3D shape-based and electrostatic similarity searches, and docking-based VS. Briefly, the retrospective study encompassed three distinct targets from diverse protein classes, each exhibiting varying degrees of active-site flexibility. Approximately 1000 structurally diverse, experimentally verified, active and inactive compounds against each of the three targets were embedded in a decoy set of 500 K molecules. We evaluated each approach on enrichment of actives in the top 100 and 1000 ranked molecules, starting from a single X-ray ligand/protein structure for all three targets. The results demonstrated that GB’s QP-based VS approach enriched hit compounds at an overall hit rate of up to 1.5%. While those rates are not exceptional in themselves, almost all of the molecules that were identified belonged to chemotypes that were distinct from those retrieved by J&J’s conventional approaches, including, unexpectedly, identifying a chemotype that formed water-mediated hydrogen bond interactions not found in the starting structures. Further, GB’s method performed best when addressing targets with flexible active sites. These findings provided the rationale to advance to a prospective evaluation of GB’s platform on the Dengue NS5 RdRP which presents challenging and highly flexible binding sites. While we provide high-level retrospective benchmarking information here for context, detailed software vendor-specific outputs and proprietary chemotypes cannot be disclosed due to standard licensing and IP obligations in pharmaceutical R&D; importantly, all claims in this work are grounded in the fully shared prospective experimental data.

The results from this prospective study further corroborate our retrospective findings. GB’s approach demonstrated the highest hit rate and most promising compounds against the NITD-640-binding pocket—a site characterized by high flexibility and disorder in the absence of the ligand. NITD-640 binds at the RNA template entrance in the fingers region of the polymerase, specifically engaging motif F (residues 468–473), which is disordered in the *apo* structure.^25^ Upon binding to NITD-640, residues M454–K469 become ordered. Further, Osawa et al.^25^ note that NITD-640 forms extensive interactions, notably wrapping around R457 and engaging in hydrogen bonding via its carboxyl and tetrazole groups with R457, E458, and K578. These interactions are critical for binding, as removal of the carboxyl group abolishes affinity. Additional water-mediated hydrogen bonds and hydrophobic contacts further reinforce the binding. Notably, despite the apparent importance of the tetrazole and carboxyl groups in NITD-640s binding, neither moiety is present in the hits identified by GB’s method, underscoring its ability to uncover novel chemotypes that are highly divergent from the reference ligand.

Since the quantum pharmacophore approach operates in a topological rather than Cartesian coordinate space, any inferred binding poses or structural hypotheses are necessarily exploratory and may risk overinterpretation. To avoid attributing unwarranted precision to pose predictions, we have not included binding-mode models in the manuscript; these can be shared upon request.

Importantly, while traditional structure-based workflows often rely on explicit binding poses and MD simulations to resolve atomic-level interactions and guide medicinal chemistry, the QP framework provides this guidance through a fundamentally different, topology-driven mechanism that does not depend on a single assumed Cartesian binding mode. Once computed and projected into Cartesian space, the QP features can be displayed as ‘hotspots’ on *any* molecular isodensity surface. This is illustrated in Supplemental Figure 3, where hotspots are rendered on an arbitrary ligand surface to provide a visual guide for medicinal chemistry optimization, helping to identify structural features potentially important for maintaining target affinity while revealing peripheral regions that may tolerate substitution or scaffold modification. Importantly, since QTAIM rigorously partitions Cartesian space into atomic basins, QP features can be decomposed and attributed to individual atoms, enabling a quantitative SAR optimization framework at atomic resolution. Therefore, the design-relevant insight typically sought from MD simulations is inherently embedded within the QP framework itself. In this sense, the QP framework replaces pose-dependent interpretability with interaction-centric interpretability.

The systematic implementation of VS via QPs delivers multiple theoretical and practical advantages. Beyond improving accuracy and enabling the identification of structurally diverse hits, it also offers substantial computational efficiencies and, as noted earlier, can be executed on standard hardware. These algorithmic advantages are further amplified in GB’s synthon-based procedure for the generation of structurally novel molecules (not employed in this work, but available for future studies). The synthon-based procedure leverages the approximate additivity of partial pharmacophores into combinatorial growth, which results in additional acceleration of multiple orders of magnitude. Due to the localized nature of the quantum interactions, the pharmacophore features present on individual synthons are mostly preserved on the resulting new synthetic product. This allows for rapid investigation of vast chemical spaces through combinatorial growth. To span considerably larger sets of potential bioactive molecules, GB’s method uses a quantum evaluation of novel compounds that can be synthesized via routine single-step chemical reactions from readily available reagents (synthons). Combinatorial databases of chemical compounds provide a substantial structural advantage since a relatively small number of synthons (e.g., ∼1 million) can result in a huge set (1 million × 1 million = 1 trillion) of diverse chemicals. The procedure starts with a quantum screening of a database of nearly 2 million synthons. Since localized quantum attributes are approximately additive, this allows for fast QP based search that can be filtered to a radically smaller compound library for subsequent 3D enumeration. This focused library is then prioritized by the complete quantum scores with high enrichment factor. This approach enables scalable exploration of large chemical spaces while incorporating synthesizability constraints, conceptually analogous—though entirely in silico—to DNA-encoded library (DEL) methodologies. Although not utilized in this work, this synthon-based approach provides a natural extension for future screening efforts across this or other targets.

In conclusion, this work has identified hits that span a range of biochemical potencies, with IC50 values from low single-digit micromolar to ∼100 μM across the three binding sites, while maintaining favorable ligand efficiency and drug-like properties. These results demonstrate that the QP-based approach can identify chemically diverse starting points with meaningful biological activity, even at challenging and highly dynamic binding sites. We believe these compounds present promising starting points for further optimization in a medicinal chemistry campaign. Owing to internal reprioritization, funding for the dengue NS5 program was suspended, and efforts to advance structural determination (through X-ray crystallography or cryo-EM) or hit optimization were deprioritized. Nevertheless, in the spirit of open science and collaborative innovation, we are releasing these set of validated hits to accelerate community-driven drug discovery against dengue and other mosquito-borne flaviviruses that pose global health challenges.

## Data and Software Availability

The cocrystal structures used for QP modeling (PDB IDs: 5JJR, 6XD0, and 6XD1) are publicly accessible via the Protein Data Bank (https://www.rcsb.org/). The chemical library employed for VS was the Enamine REAL Diverse drug-like set, version dated February 2023. Updated versions of this library are available at https://enamine.net/compound-collections/real-compounds/real-database-subsets. Chemical structures of the experimentally confirmed hits are presented in Figure 5.

The source code utilized in this study is proprietary to GB and developed by MNM. While it is not publicly available, MNM is open to collaborative engagements that support scientific advancement and reproducibility. Interested parties are encouraged to contact MNM to explore potential avenues for collaboration under appropriate agreements.

## Methods

### Cells, Viruses and Compounds

African green monkey kidney cells (Vero; CL 84113001, European Collection of Authenticated cell cultures (ECACC) were maintained in Eagle’s minimal essential medium (MEM; Gibco) supplemented with 10% (v/v) FBS (Biowest), 2 mM Alanyl-Glutamine (Sigma) and 0.02 mg/mL gentamicin (Gibco). Huh7 hepatoma-derived cells (Sigma) were maintained in Dulbecco’s modified Eagle’s medium (DMEM, Gibco), supplemented with 10% FBS, 2 mM Ala-glutamine, 1 mM sodium pyruvate (Gibco) and 0.02 mg/mL gentamicin. All cells were cultured at 37 °C with 5% CO_2_ in a humidified incubator. All cell lines were regularly tested for mycoplasma contamination.

DENV-2/16681-eGFP, carrying an enhanced green fluorescent protein (eGFP) at the amino terminus of the capsid protein, was produced by transfection of in vitro-transcribed RNA of plasmid pFK-DV-G2A into Huh7 cells.^58^

The enamine REAL diversity library containing 43.8million compounds was used to perform the quantum similarity searches. Individual compounds purchased from Enamine were solubilized as 10 mM stocks in 100% DMSO and stored at −20 °C until use.

The reference compounds used herein, compound 29,^28^ NITD-434, and NITD-640,^27^ were synthesized in J&J and stored as 10 mM stocks in 100% dimethyl sulfoxide (DMSO) at −20 °C.

### Protein Expression and Purification

The DENV2 NS5 full length protein (New Guinea C, AAC59275.1) and DENV3 NS5 (Sri Lanka, YP_001531176.2) were subcloned into pVL1393 vector with C-terminal 8xHis-tags. For protein expression, *T.ni* (High Five) cells were infected with 10%(v/v) virus and grown for 48 h before harvesting by centrifugation. Cell pellets were resuspended in lysis buffer (20 mM Tris, pH 7.5, 300 mM NaCl, 10% Glycerol, 1 mM TCEP, and 10 mM imidazole), supplemented with 1 mM PMSF and 50 μL/L Benzonase Nuclease and lysed by sonication. Lysate was clarified by centrifugation and incubated with pre-equilibrated Ni-NTA resin for 1 h. Protein was eluted using a stepwise elution protocol, increasing imidazole concentration to 500 mM. Fractions containing DENV protein were pooled, concentrated and loaded onto a Superdex 200 column (Cytiva) equilibrated in 20 mM Tris, pH 7.5, 300 mM NaCl, 5% Glycerol, 1 mM TCEP. Peak fractions containing DENV protein were pooled and concentrated to 7.46 mg/mL (70 μM) and 8.43 mg/mL (79.6 μM), DENV2 NGC NS5 and DENV3 SL NS5, respectively. Protein purity and homogeneity were evaluated by SDS-PAGE, intact Mass spectrometry and analytical SEC.

DNA fragments encoding DENV2_PG NS5 RdRp residues 1–901 (Papua New Guinea, ACQ44517) were amplified via PCR and subcloned into the pFBD-BirA expression vector. The insert was positioned downstream of the AviTag for in vivo biotinylation and upstream of a HisTag. The resulting plasmid was transformed into DH10Bac Competent *E. coli* (Invitrogen) to generate recombinant viral DNA bacmids, which were purified and used to produce recombinant baculovirus in Sf9 insect cells. Cells were harvested by centrifugation at low speed (2500 rpm for 10 min at 4 °C in a Beckman Coulter centrifuge) when cell viability dropped to 70–80%. The cells were resuspended in extraction buffer (20 mM Tris-HCI, pH 7, 500 mM NaCl, 5% glycerol, 5 mM Imidazole) + 1 mL protease inhibitor (PI) cocktail (Aprotinin, Leupeptin, Pepstatin A, and E-64) and lysed chemically by adding NP40 (final concentration of 0.5%), 1 mM TCEP, 1 mM PMSF/Benzamidine HCl and 20 μL/L Benzonase Nuclease (in-house), followed by sonication at the frequency of 7.0 (10″ on/10″ off) for 5 min (Sonicator 3000, Misoni). The crude extract was then clarified by high-speed centrifugation (60 min at 36,000 × *g* at 4 °C) in a Beckman Coulter centrifuge to remove cellular debris. The clarified lysate was first loaded onto a Ni-NTA resin column, followed by Gel filtration using a Superdex 200–26/600 (Cytiva) in 50 mM Tris, pH 7, 500 mM NaCl, 5% glycerol and 1 mM TCEP, to enrich DENV RdRp NS5 to 95% purity. Following the identification of protein eluting fraction and purity using SDS-PAGE gels, and mass confirmation, the fractions were pooled, concentrated, snap-frozen, and stored at −80 °C until use in SPR. Protein mass was confirmed by LC-MS.

Expression and purification of human mitochondrial DNA-dependent RNA polymerase (h-mtRNAP, NP_005026.3) and DENV2_TSV01 (Townsville, AY037116.1) polymerases were conducted as previously described using the pFastBac-1 plasmid. The purified protein was stored in 100 mM Tris pH 7.5, 100 mM NaCl, 40% glycerol, 5 mM TCEP, 0.01% Tween-20 and stored at −80 °C until use in the RNA synthesis assay.

### Calculation of QPs

QP generation is exemplified using the 5JJR model, based on DENV3 NS5 cocrystallized with inhibitor compound 29 (Figure 2). Using structurally resolved atomic coordinates as modeling data, the supermolecular ligand-target complex is rigorously partitioned into subsystems (eq 4, Figure 2A). The energy components are computed and presented as a QP of the interaction of compound 29 and the target (Figure 2B). The QP model was used for fast comparison (screening) to other potential ligands with similar QPs that are chemically different from the cocrystallized one and may possess better pharmacological properties.

### VS Results and Compound Selection

Three QP models were generated using PDB structures 5JJR, 6XD0 and 6XD1, cocrystallized with RdRP domain inhibitors Compound 29,^28^ NITD-434 and NITD-640,^27^ respectively. The models were validated and used for VS of Enamine REAL diverse drug-like library version 02.2023 (∼43.8million compounds).^59^ The library satisfies Lipinski’s Ro5 and Veber criteria: MW ≤ 500, *S*log*P* ≤ 5, HBA ≤ 10, HBD ≤ 5, RotBonds ≤ 10, and TPSA ≤ 140 and lacks undesirable chemical groups such as PAINs.

### NS5 Elongation Assay

The initial hit selection and subsequent concentration dependent curves for the virtual screen hits were determined using the NS5 elongation assay against DENV2 NGC, DENV3 SL. Compounds were tested in concentration series starting at 200 μM, in a 1:4 dilution series.^60^ To determine their effect on the polymerase activity, the following assay conditions were used: for DENV2 NGC and DENV3 SL, 2× enzyme stocks were prepared in assay buffer containing final concentrations of 20 nM enzyme, 25 mM Tris pH 7.5, 2 mM MnCl_2_, 3 mM MgCl_2_, 0.01% Pluronic F-127, 7.5% glycerol, 1 mM DTT, 0.1% Prionex, 0.25 mM EGTA and 1% DMSO. The enzyme solution was dispensed in a 384-well plate, followed by a 1 min centrifugation at 1500 rpm. Immediately after, a substrate solution containing 50 nM PolyC and 35 μM GTP final in assay buffer, is added to the each well, followed by centrifugation for 1 min at 1500 rpm. The reaction was incubated for 3 h at room temperature. Next, the polymerase activity was quenched using 20 mM EDTA in assay buffer and detected using 0.11% Quant-iT PicoGreen. Fluorescence intensity was measured using a Pherastar FSX with excitation at 485 nm and emission at 520 nM (5 flashes). The raw data was normalized and IC_50_ values were determined using Genedata Screener.

### Surface Plasmon Resonance

Binding affinity of the compounds was tested by SPR using Biacore 8K (Cytiva) instrument at 20 °C. Biotinylated DENV RdRp ns5 (1–901) protein was immobilized onto the active flow cell of the SA chip following the manufacturer’s protocol reaching approximately 7000 response units (RU). For compounds whose binding profile showed protein conformational changes, a lower protein level (2000–3500 RU) was immobilized to confirm the *K*_D_ reproducibility. Compounds were initially dissolved in 100% DMSO and serially diluted (factor: 0.5 or 0.3) to obtain seven concentrations points in 100% DMSO. For the SPR run, the DMSO stocks were diluted 1:50 in the assay buffer (20 mM HEPES, pH 7.4, 150 mM NaCl, 0.005% Tween20 (v/v)) supplemented with 0.5 mM reducing agent (TCEP) to achieve a final concentration of 2% DMSO. The tested compounds were injected over the reference and active flow cells using multicycle kinetics with a contact time of 60 s (300 s for Flavi_005409, Flavi_005462, Flavi_005533 and 600 s for Flavi_005318) and a dissociation time of 120 s (600 s for Flavi_005409, Flavi_005462, Flavi_005533 and 1200 s for Flavi_005318) at a flow rate of 40 (20 and 10) μL/min at 20 °C. Additionally, a 5-point solvent correction was included for each run to adjust high bulk responses from the solvent. Double referencing of the data was performed by subtraction of the reference flow cell and then the respective blank cycles. Data analysis was performed to obtain the best fit for each tested compound using Biacore Insight evaluation software. Steady-state fitting, in addition to kinetic fitting for compounds Flavi_005318, Flavi_005409, Flavi_005451, Flavi_005462 were performed by applying 1:1 binding or two-state reaction model provided by the software. The final figures were prepared in the GraphPad Prism version 9.4.1

### Dynamic Light Scattering (DLS)

The aggregation of compounds was estimated by DLS, which directly measures compound aggregates and laser power in solutions. Compounds were prepared at 15 mM, 7.5 mM and 3.75 mM directly from DMSO stocks, then diluted 50x into filtered 20 mM HEPES, pH 7.4, 150 mM NaCl, 0.005% Tween20, 0.5 mM TCEP (2% DMSO final). The resulting samples were then distributed into 384-well plates (Corning, Cat# 3540), with 20 μL in each well. The sample plate was centrifuged at 3500 rpm for 5 min before loading into DynaPro DLS Plate Reader III (Wyatt Technology). The results can be found in Supplementary Table 2.

### RNA Synthesis Assay (Gel-Based)

h-mtRNAP DNA-dependent RNA polymerase assay was adapted with minor modifications from Lu et al.^61^ DENV2_TSV01 NS5 polymerase de novo RNA synthesis assay involved a 20 min preincubation of 0.5 μM polymerase with a range of inhibitor concentrations from 1.2 to 1000 μM or DMSO (10%) in the presence of 5 mM MgCl_2_ at 30 °C. The RNA synthesis was initiated by adding 2 μM RNA template (20 nucleotides from the 3′-end of the genome, Horizon Discovery, USA), 500 μM ATP, GTP, CTP mix, and 0.1 μM [α-^32^P]-UTP (∼1.2 μCi, Revvity, USA). Reactions (15 μL) were incubated for 20 min at 30 °C and then stopped by the addition of 45 μL of formamide/EDTA (25 mM) mixture and incubated at 95 °C for 10 min. Three μL reaction samples were subjected to denaturing by 8 M urea 20% polyacrylamide gel electrophoresis to resolve products of RNA synthesis, followed by signal quantification (ImageQuant 5.2, GE Healthcare Bio-Sciences, Sweden) through phosphor imaging (Amersham Typhoon 5, Cytivia, USA).

### Antiviral Assays

The in vitro antiviral activity of the selected hits was assessed in two cell lines, Vero and Huh7, against DENV2-eGFP, following published methods.^58,61^ Briefly, Vero or Huh7 cells were seeded in 384-well black view plates containing 200 nL of compounds in a 9-point serial dilution. The next day, DENV2/16681-eGFP at an MOI of 0.5 (Vero) or 5 (Huh7) was used for infection, including virus control. No virus inoculum was added to the cell control. After 3 days, the eGFP expression was quantified using an EnVision Microplate Reader (PerkinElmer). Cell viability or cytotoxicity of the compounds was determined by addition of ATPlite reagent (PerkinElmer) to all wells. After a 10 min incubation, the plate was read out for luminescence with an EnVision. The EC_50_ and CC_50_ were calculated using Genedata Screener.

## Supplementary Material



## Notes

DENV2 SPR results; dose–response SPR binding profiles for 5 hit compounds; aggregation of compounds measured by DLS; dose response curves for all hits in Vero and Huh7 cell lines; and interaction hotspot surface (PDF)
