# Supplementary material for: Quantum Pharmacophore-Based Virtual Screening Enables Prospective Discovery of Chemotype-Diverse Dengue NS5 Inhibitors
Source: J Chem Inf Model. 2026 Jul 16;66(14):8304–17. doi: 10.1021/acs.jcim.6c01051 (PMC13417884; doi:10.1021/acs.jcim.6c01051)
Supplement: Supplementary file 1 [file ci6c01051_si_001.pdf]

Supplementary Information

Quantum Pharmacophore-Based Virtual Screening Enables Prospective Discovery of Chemotype-diverse Dengue NS5 Inhibitors

Martin N Martinov[1,2*$] and Annelies Van Den Bergh[3$], Edgar Jacoby[3], Ivaylo Kirov[4], Lyubomir G Nashev[4], Oleksandra Herasymenko[5], Cheryl H Arrowsmith[5], Jeffrey W Slater[6], Tsehai Grell[6], Kenneth Maksimchuk[6], Michael D Hack[7], Zhe Wu[6], Donya Ohadi[6], Doortje Borrenberghs[3], Marnix Van Loock[3], Chandrika Mulakala[8*] and Olivia Goethals[3]

1 Gradient Biomodeling, Park City, Utah, 84098, USA
2 FAR Biotech, Madison, WI 53719, USA
3 Johnson & Johnson Research and Development, Beerse, Antwerp 2340, Belgium
4 Gradient Bulgaria, Sofia 1164, Bulgaria
5 Structural Genomics Consortium, University of Toronto, Toronto, ON M5G 1L7, Canada
6 Johnson & Johnson Research and Development, Spring House, PA 19477, USA
7 Johnson & Johnson Research and Development, San Diego, CA 92121, USA
8 Johnson & Johnson Research and Development, South San Francisco, CA 94080, USA
$ M.N.M and A.V.D.B contributed equally to this work
* Corresponding authors: Chandrika Mulakala, Email: cmulakal@its.jnj.com; Martin N Martinov, Email: martinov.mn@gradientbiomodeling.com

Supplementary Table 1. DENV 2 SPR results.

Pocket	Compound	**SPR**			
		DENV2_PG NS5 RdRP N=2-4			
		K_D (μM)	R_{max} (RU)	k_a (1/Ms)	k_d (1/s)
5JJR	JNJ-8284	5.5 ± 1.3	177	1146/0.029	0.062/0.0048
	Compound 29	0.1 ± 0.05	40.9	/	/
6XD0	JNJ-3465	22.6 ± 7.2	41.7	/	/
	NITD-434	17.8 ± 8.4	88.2	/	/
6XD1	JNJ-2763	34 ± 4	74.9	141/1.98E-07	0.0044/0.002
	JNJ-2750	Linear response	/	/	/
	JNJ-2594	10 ± 2	249	448	0.0033
	NITD-640	12.0 ± 8.5	192	/	/

k_a and k_d are provided for 1:1 binding kinetic model, and k_{a1}/k_{a2} and k_{d1}/k_{d2} are provided for a two-state reaction kinetic model

Supplementary Figure 1. Dose-response SPR binding profiles for 5 hit compounds. (a) SPR sensorgram with kinetic fit using 1:1 binding model. (b) SPR sensorgram with kinetic fit using two state reaction model. (c) SPR sensorgram and plot with steady state affinity fit using 1:1 binding model.

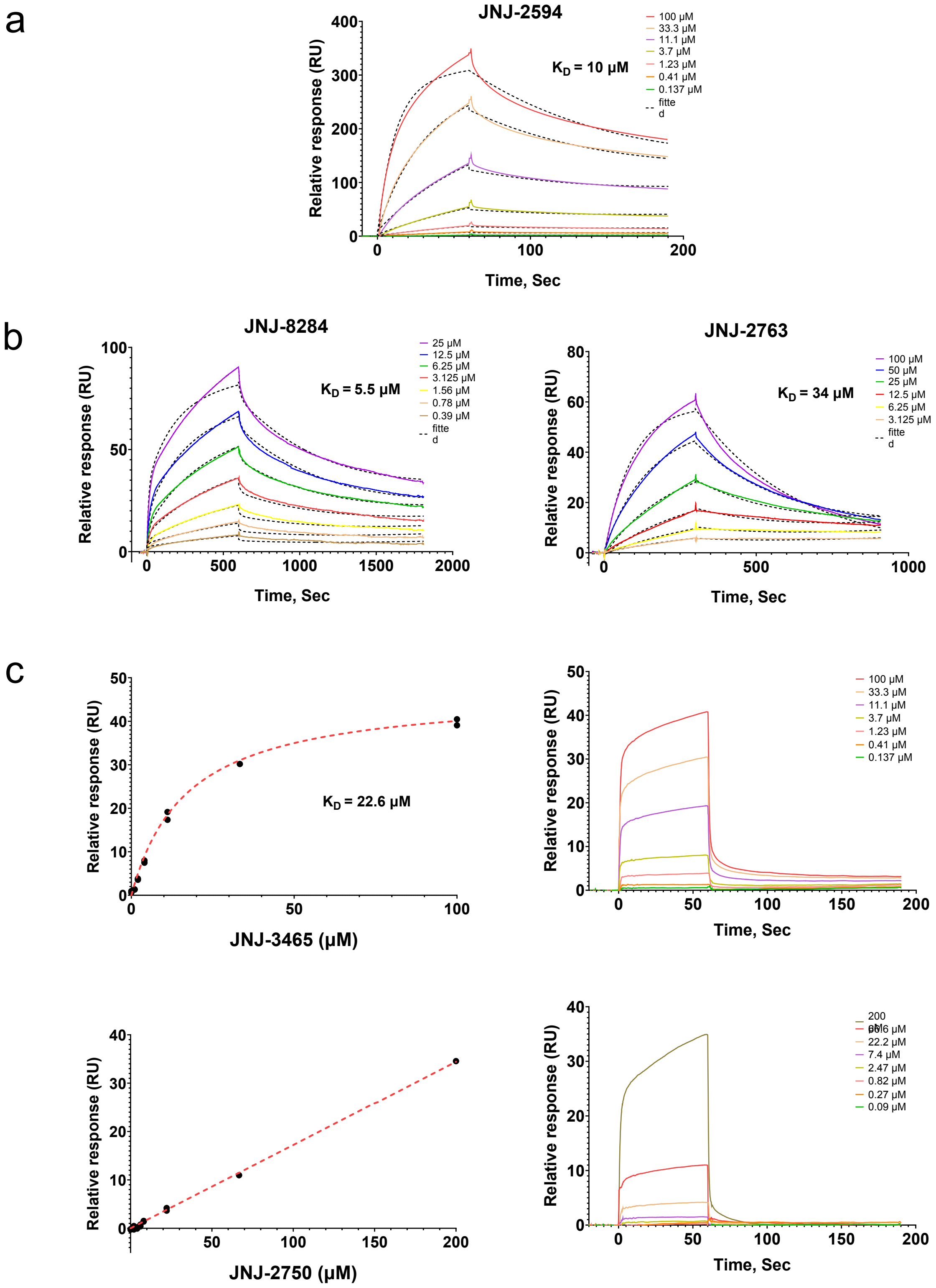

Supplementary Table 2. Aggregation of compounds measured by DLS. Values highlighted in red indicate aggregation of the compound at the respective concentration.

Compound ID	Intensity (kCnt/s)				% Laser Power			
	0 µM	50 µM	150 µM	300 µM	0 µM	50 µM	150 µM	300 µM
JNJ-2763	974	656	1992	64350	100	100	73	20
NITD-434	825	538	709	1049	100	100	100	90
JNJ-3465	610	763	1743	9602	100	100	45	100
Compound 29	686	645	4718	1812	100	100	30	53
JNJ_8284	545	611	3731	33044	100	100	63	38
JNJ-2750	907	479	3912	115934	100	100	60	18
JNJ-2594	1167	622	816	1935	100	100	100	63

Compound ID	Intensity (kCnt/s)				% Laser Power			
	0 µM	33.3 µM	100 µM	200 µM	0 µM	33.3 µM	100 µM	200 µM
NITD-640	919	653	711	567	100	100	100	100

Supplementary Figure 2. Dose response curves for all hits in Vero and Huh7 cell lines. Individual data points represent the mean of at least three experimental repeats.

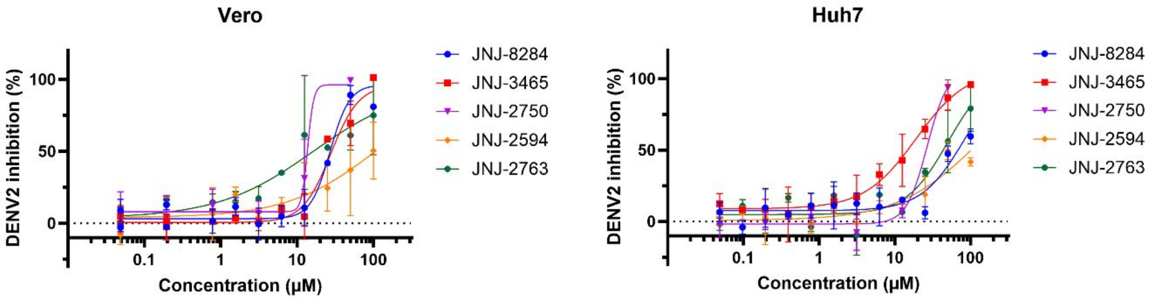

Supplementary Figure 3. The interaction hotspot surface (blue, reduced solvent radius of 0.5 Å) directly highlights the spatial regions important for the interaction. Surface hotspots emphasize the chemically relevant portions of the ligand that contribute most strongly to binding, helping to distinguish essential pharmacophoric regions.

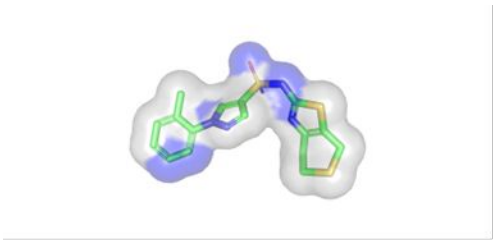

JNJ-8284

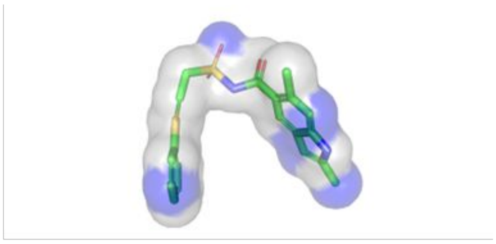

JNJ-3465

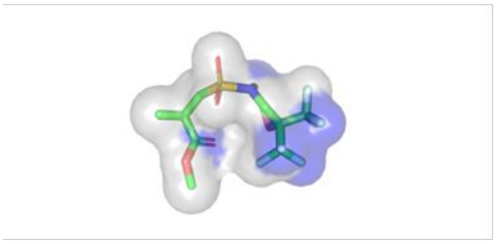

JNJ-2594

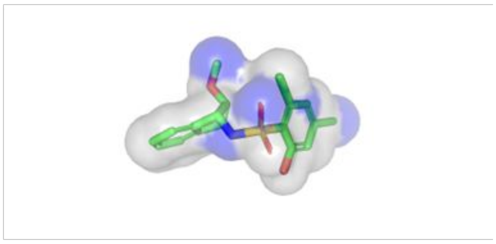

JNJ-2750

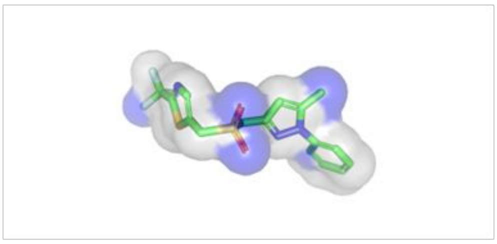

JNJ-2763
